# Dermatosis neglecta in a case of multiple fractures, shoulder dislocation and radial nerve palsy in a 35-year-old man: a case report

**DOI:** 10.1186/1752-1947-2-347

**Published:** 2008-11-17

**Authors:** Syed Nurul Rasool Qadir, Amer Ejaz, Naeem Raza

**Affiliations:** 1 Skin department, Combined Military Hospital, Kharian Cantt, 75500, Pakistan

## Abstract

**Introduction:**

Dermatosis neglecta is an often misdiagnosed and under-diagnosed condition. In dermatosis neglecta, a progressive accumulation of sebum, sweat, keratin and other dirt and debris, occurs due to inadequate local hygiene resulting in a localized hyperpigmented patch or a verrucous plaque. Vigorous rubbing with alcohol-soaked gauze or soap and water results in a complete resolution of the lesion. This is the first case of dermatosis neglecta reported in a patient with multiple traumatic injuries.

**Case presentation:**

We report a case of a 35-year-old male Caucasian of Pakistani origin, with multiple fractures, neurological deficit and immobility sustained in a fall, leading to the development of dermatosis neglecta of the left hand.

**Conclusion:**

Early and prompt clinical recognition of this condition eliminates the need for aggressive diagnostic and therapeutic procedures.

## Introduction

The term dermatosis neglecta was first coined by Poskitt et al. in 1995 to denote a condition in which formation of a localized hyperpigmented lesion occurs as a consequence of lack of cleanliness of a particular body part or region, usually due to some disability [1].

The lesion forms due to a combination of tallow, sebum, sweat, keratin and bacteria in the unclean area. The time of evolution is usually 2 to 4 months and the patients usually have an associated chronic disease characterized by pain or immobility [2]. Rubbing with alcohol-soaked gauze or washing with soap and water causes the lesion to completely disappear. The result of treatment usually surprises patients who may initially be reluctant to admit that the condition is due to negligence.

## Case presentation

**Figure 1:**
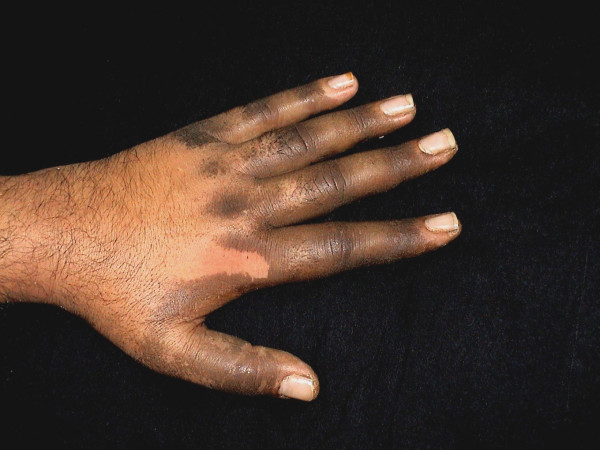
Dorsum of hand (at presentation).

A 35-year-old man presented with a 3-week history of progressive blackish discoloration of the dorsum of the left hand along with increased verrucosity and scaling over his palm (Figure 1). There was also moderate pruritus over the affected area. No other body part was involved and there were no systemic symptoms.

The patient had sustained multiple fractures of the left humerus and metacarpals, along with dislocation of the left shoulder and radial nerve palsy, in a fall about 2 months previously which had left the limb immobile and numb. He was gradually recovering the motor and sensory functions but still handled the limb very gingerly.

**Figure 2:**
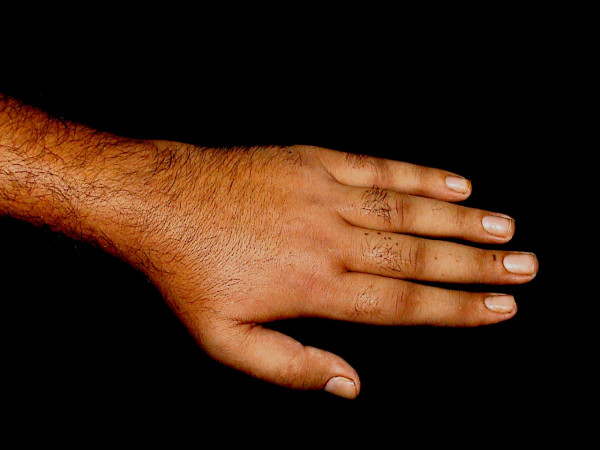
Dorsum of hand (after two weeks).

Considering the history and clinical examination, a diagnosis of dermatosis neglecta was made. The area over the dorsum of the hand was cleaned with a methanol swab, revealing completely normal skin underneath. The patient was prescribed a keratolytic ointment for the palmer surface and advised to maintain better hygiene of the affected area despite the disability. Upon follow-up examination two weeks later, the hand was completely devoid of any pigmentation or verrucosity (Figure 2).

## Discussion

The term dermatosis neglecta is used to describe a condition in which localized hyperpigmentation and scaling of the skin occurs as a consequence of poor hygiene of a particular body part and the lesion can be easily rubbed off using soap and water or an alcohol soaked swab. The lack of cleanliness is usually a result of hyperesthesia or prior trauma of the affected region [1,3]. Terra firma forme dermatosis has also been used to describe a condition with similar clinical features but which is not amenable to soap and water cleansing and can only be rubbed off with vigorous alcohol swabbing [4-6]. It is in all probability a more severe variant of dermatosis neglecta rather than a separate clinical entity.

Clinically, the patient presents with a hyperpigmented patch or plaque with a variable degree of scaling and verrucosity. Adherent cornflake-like scaling has been described [7]. The pathogenesis centres on insufficient exfoliation in a particular area leading to accumulation of corneocytes, sebum, sweat and bacteria. The longstanding asymptomatic accumulation of dirt may lead to verrucous plaques simulating verrucous naevi [3]. Pityrosporum orbiculare has been isolated from some lesions but may represent yeast overgrowth in a conductive environment rather than a causative factor [7].

Cases have previously been described at the site of pacemaker insertion, mastectomy surgery and radiotherapy [7]; and in dermatomyositis, hemiplegia and keloidal scars [2], as well as in the periareolar region bilaterally [3], but to our knowledge this is the first case report in a patient with multiple traumatic injuries.

Differential diagnosis includes dermatitis artefacta which is an act of commission rather than an act of omission as is the case in dermatosis neglecta [1], verrucous naevi [3], acanthosis nigricans, Vagabond's disease, hyperkeratotic Malassezia dermatosis [8] as well as confluent and reticulated papillomatosis of Gougerot and Carteaud, frictional asymptomatic darkening of the extensor surfaces, idiopathic deciduous skin and post-inflammatory hyperpigmentation [7].

Treatment includes counselling and encouraging the patient to maintain appropriate hygiene of the affected region in spite of his or her disability. Daily lightly scrubbing of the affected area with soap and water or alcohol is effective in most cases. For more resistant and verrucous lesions, application of a keratolytic agent in combination with an emollient may be required.

## Conclusion

Dermatosis neglecta should be kept in mind in the differential diagnosis of all hyperpigmented localized lesions, especially in a patient with some accompanying disability, as its prompt recognition can eliminate the need for any elaborate diagnostic or therapeutic endeavours.

## Consent

Written informed consent was obtained from the patient for publication of this case report and any accompanying images. A copy of the written consent is available for review by the Editor-in chief of this journal.

## Competing interests

The authors declare that they have no competing interests.

## Authors' contributions

SNRQ conceived the case report and prepared the initial draft of the manuscript. AE played an important role in the initial diagnosis, treatment and follow-up of the patient as well as in writing the manuscript. NR contributed significantly to the final draft of the manuscript and analysis of relevant literature. All authors read and approved the final manuscript.
